# Supplementary material for: Unraveling a bifunctional mechanism for methanol-to-formate electro-oxidation on nickel-based hydroxides
Source: Nat Commun. 2023 Mar 27;14:1686. doi: 10.1038/s41467-023-37441-9 (PMC10042884; doi:10.1038/s41467-023-37441-9)
Supplement: Supplementary file 1 — Supplementary Information [file 41467_2023_37441_MOESM1_ESM.pdf]

Supplementary Information for

Unraveling a bifunctional mechanism for methanol-to-formate electro-oxidation on nickel-based hydroxides

Botao Zhu[1], Bo Dong[1], Feng Wang[1], Qifeng Yang[1], Yunpeng He[1], Cunjin Zhang[2], Peng Jin[2]*, Lai Feng[1]*

[1]Soochow Institute for Energy and Materials Innovation (SIEMIS), School of Energy, Soochow University, Suzhou 215006, China

[2]School of Materials Science and Engineering, Hebei University of Technology, Tianjin 300130, China

*Corresponding authors:

Email: fenglai@suda.edu.cn (L. Feng); china.peng.jin@gmail.com (P. Jin)

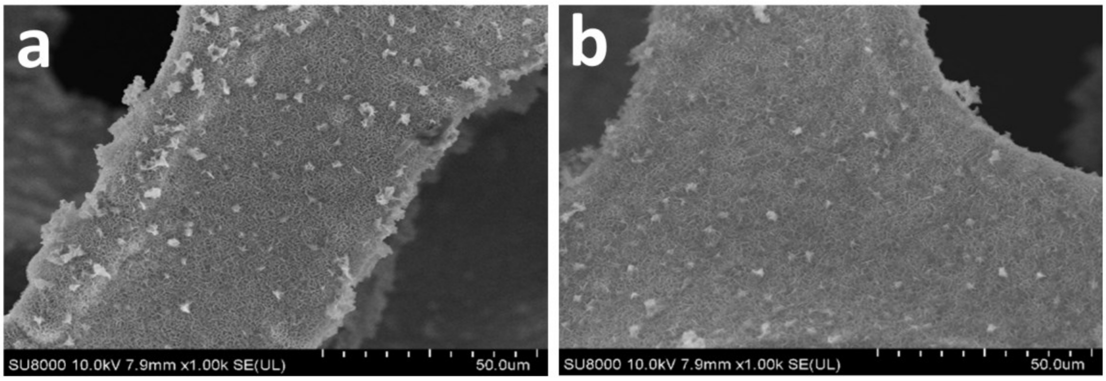

Supplementary Fig. 1. SEM images of the as-synthesized (a) NiMn-LDH/NF and (b) NiFe-LDH/NF.

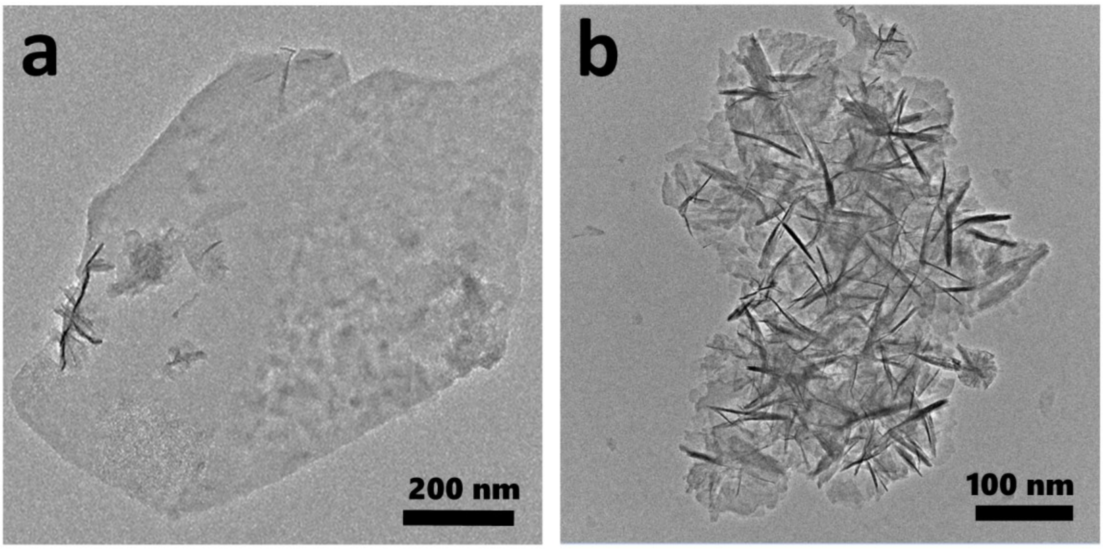

Supplementary Fig. 2. TEM images of the as-synthesized (a) NiMn and (b) NiFe-LDH.

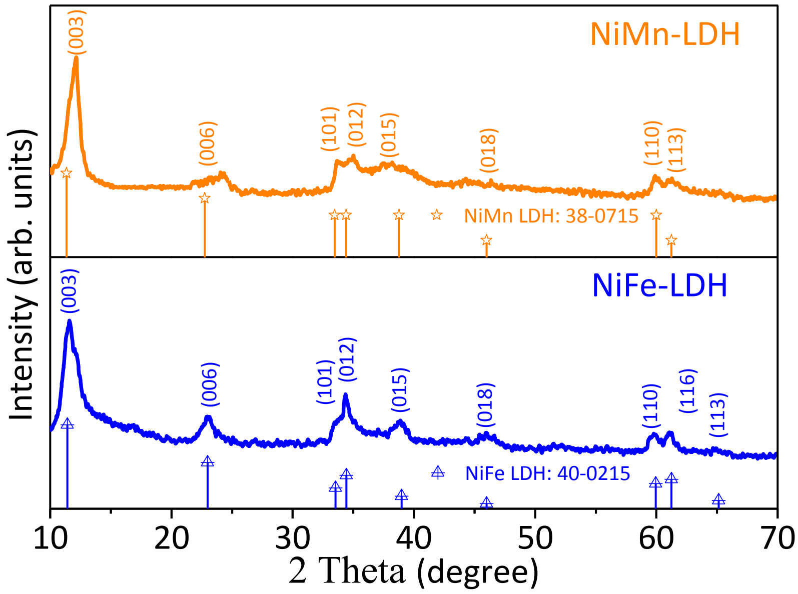

Supplementary Fig. 3. XRD patterns of the as-synthesized (a) NiMn and (b) NiFe-LDH.

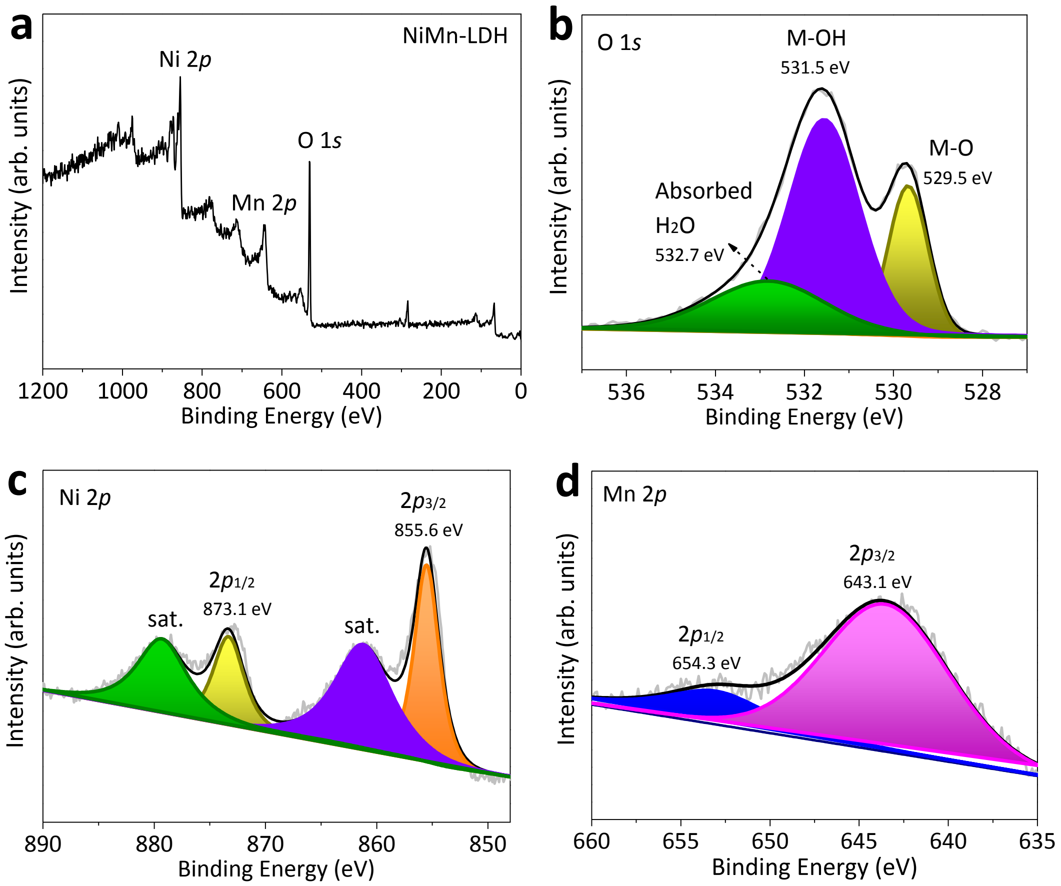

Supplementary Fig. 4. XPS spectra of the as-synthesized NiMn-LDH. (a) survey and (b) O 1*s*, (c) Ni 2*p* and (d) Mn 2*p* core-level spectra.

Supplementary note 1

The XPS survey spectra of NiMn-LDH display Ni 2*p*, Mn 2*p* and O 1*s* signals, confirming their compositions. The O 1*s* XPS spectra are fitted by three peaks, assignable to adsorbed H_2O (532.7 eV), M-OH (531.5 eV) and M-O (529.5 eV), respectively. The Ni 2*p* XPS spectra are fitted by four peaks: two spin-orbit peaks of Ni $2p_{1/2}$ (873.1 eV) and $2p_{3/2}$ (855.6 eV) corresponding to Ni^{2+} species, and two satellite peaks. The Mn 2*p* XPS spectra are fitted by two peaks: two spin-orbit peaks of Mn $2p_{1/2}$ (654.3 eV) and $2p_{3/2}$ (643.1 eV) corresponding to Mn^{3+} species. These results are in line with literature reported NiMn-LDH[1].

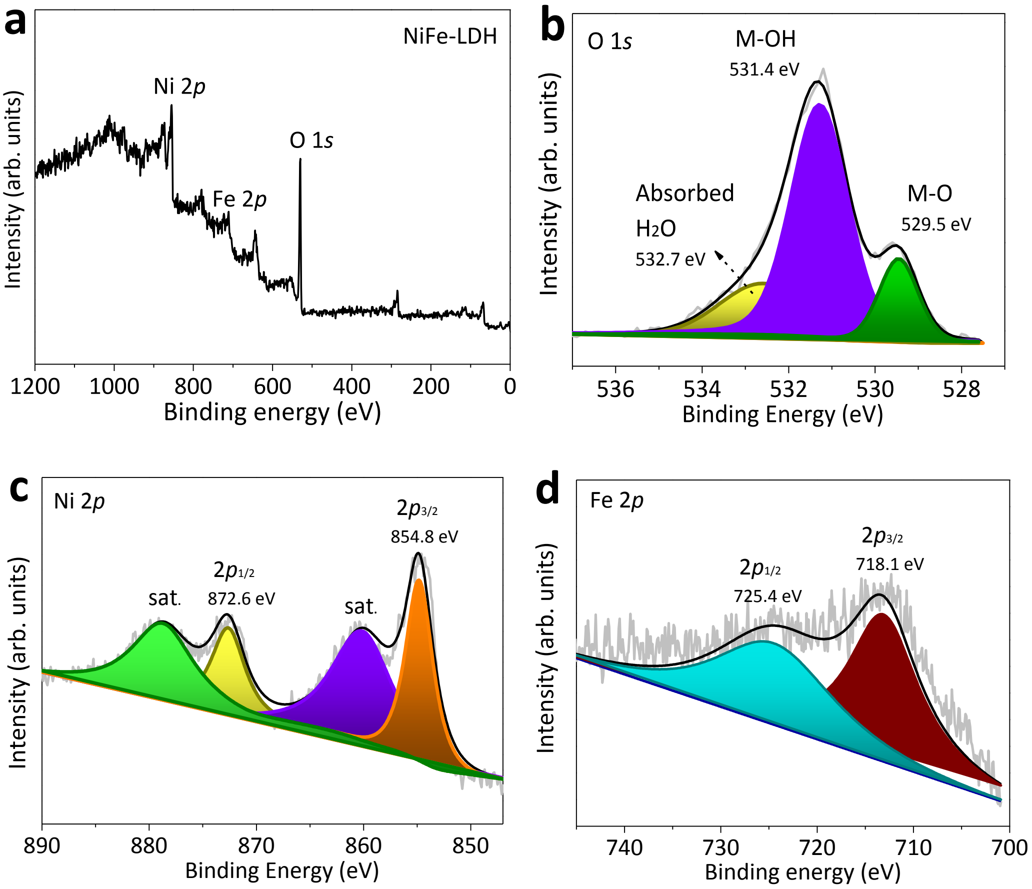

Supplementary Fig. 5. XPS spectra of the as-synthesized NiFe-LDH: (a) survey and (b) O 1*s*, (c) Ni 2*p* and (d) Fe 2*p* core-level spectra.

Supplementary note 2

The XPS survey spectra of NiFe-LDH display Ni 2*p*, Fe 2*p* and O 1*s* signals, confirming their compositions. The O 1*s* XPS spectra are fitted by three peaks, assignable to adsorbed H_2O (532.7 eV), M-OH (531.4 eV) and M-O (529.5 eV), respectively. The Ni 2*p* XPS spectra are fitted by four peaks: two spin-orbit peaks of Ni $2p_{1/2}$ (872.6 eV) and $2p_{3/2}$ (854.8 eV) corresponding to Ni^{2+}species, and two satellite peaks. The Fe 2*p* XPS spectra are fitted by two peaks: two spin-orbit peaks of Fe $2p_{1/2}$ (725.4 eV) and $2p_{3/2}$ (718.1 eV) corresponding to Fe^{3+} oxidation state. These results are in line with the literature reported NiFe-LDH[2].

Supplementary Table 1. Ni/Mn or Ni/Fe ratio calculated for as-synthesized NiM-LDH (M= Mn or Fe) based on ICP-OES analysis.

	Ni (mg/L)	M (mg/L)	Ni/M molar ratio
NiMn-LDH	23.19	4.97	4.37:1
NiFe-LDH	26.68	5.52	4.59:1

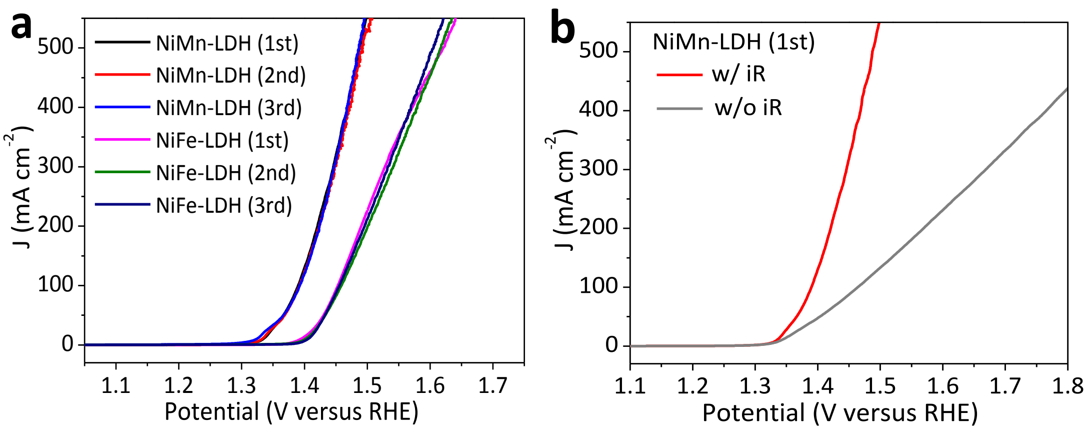

Supplementary Fig. 6. (a) LSV curves (with iR compensation) of NiMn and NiFe-LDH (including three independent electrodes per each catalyst) measured with scan rate of 5 mV s^{-1} under the MOR conditions (in 1 M KOH with 3 M methanol). (b) Comparison between the LSV curves of NiMn-LDH with and without iR compensation, where R = 1.65 Ohm.

Supplementary note 3

To explore the reproducibility of the catalyst performance, we have repeated the LSV measurements under the MOR conditions by using three independent electrodes per each catalyst. As shown in **Supplementary Fig. 6a**, the LSV curves for each catalyst are almost overlapped with each other, indicating that both the catalyst preparations and electrochemical measurements are reproducible.

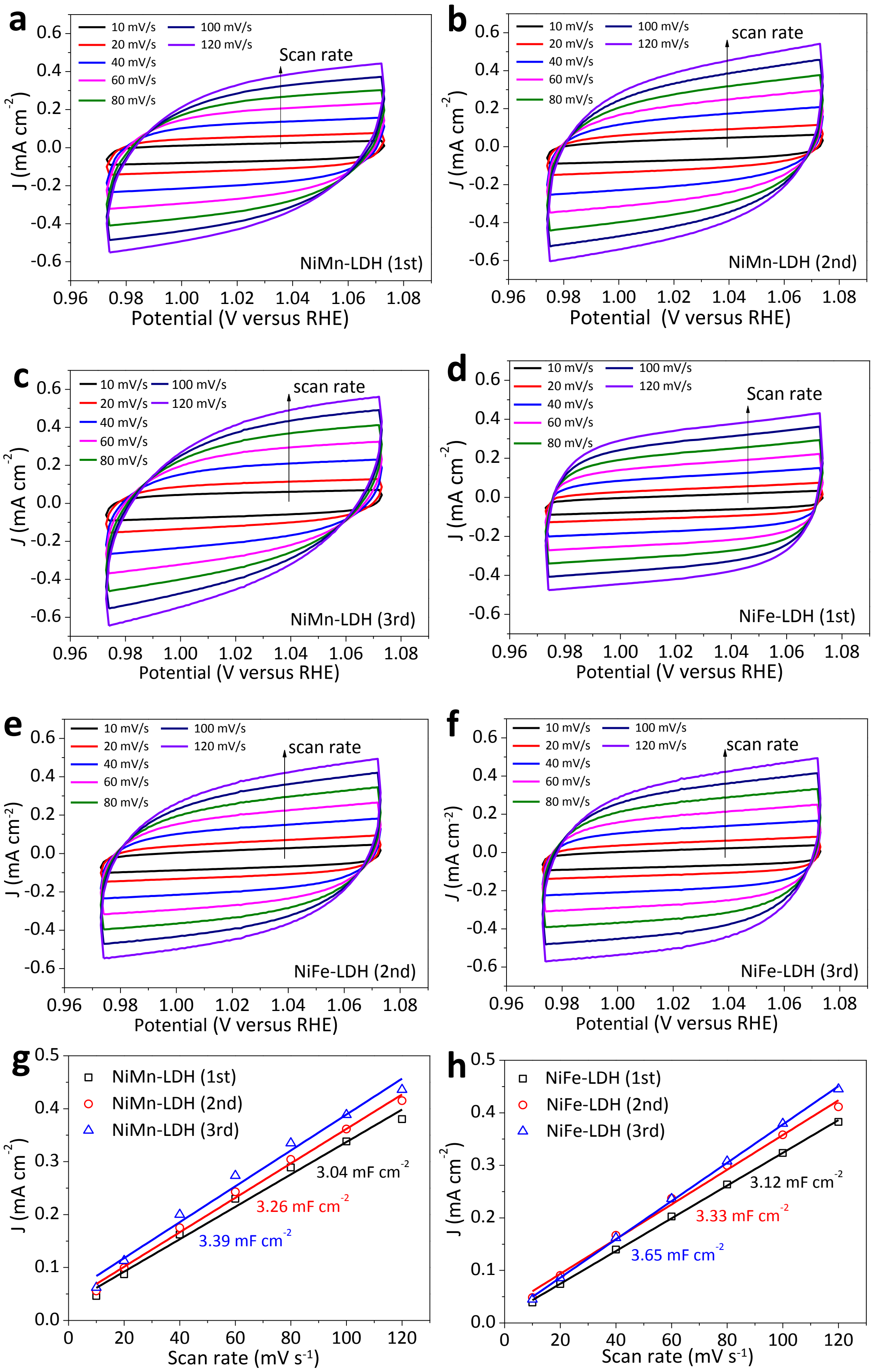

Supplementary Fig. 7. Calculation of C_{dl}: CV curves of (a-c) NiMn-LDH and (d-f) NiFe-LDH (totally three independent electrodes per each catalyst) measured in the

non-Faradaic range under the same conditions. Plots of current density and scan rate (v) of (g) NiMn-LDH and (h) NiFe-LDH. Average values of C_{dl} could be determined as 3.23 mF (NiMn-LDH) and 3.37 mF (NiFe-LDH) for the electrode with an area of 1 cm^2.

Supplementary note 4

To explore the reproducibility of the C_{dl}, we have repeated the CV measurements under the same conditions by using three independent electrodes per each catalyst. As shown in **Supplementary Fig. 7g, h**, the plots of current density and scan rate of each catalyst are very close to each other, indicating that the C_{dl} measurements are reproducible. For each catalyst, an averaged value was adopted to determine the C_{dl}.

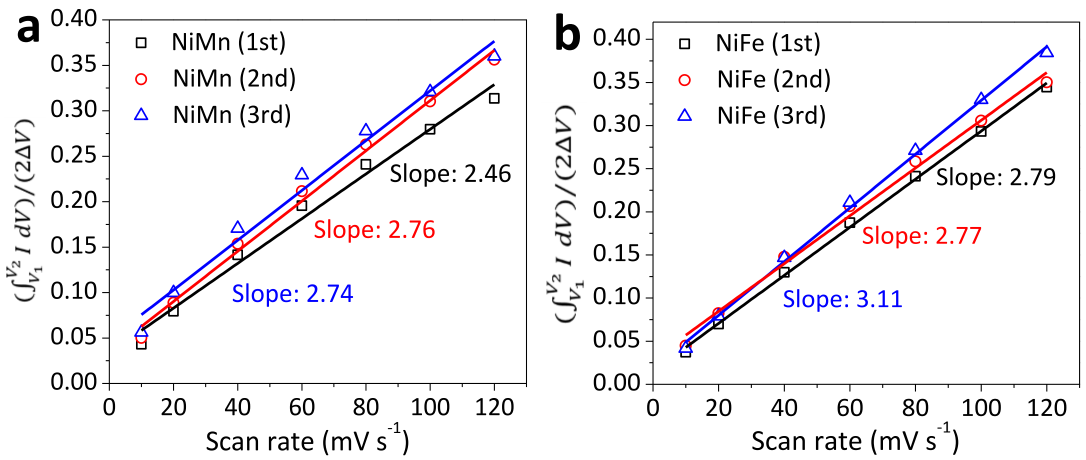

Supplementary Fig. 8. Calculation of C_s: Plots of scan rate (v) and $\frac{\int_{V_1}^{V_2} I\,dV}{2\Delta V}$ for (a) MiMn-LDH and (b) NiFe-LDH (totally three independent electrodes per each catalyst). Average values of C_s are determined as 2.65 mF cm^{-2} (NiMn-LDH) and 2.89 mF cm^{-2} (NiFe-LDH). As a result, ECSA could be determined as 1.22 cm^2 (NiMn-LDH) and 1.17 cm^2 (NiFe-LDH) based on the equation of ECSA = C_{dl}/C_s.

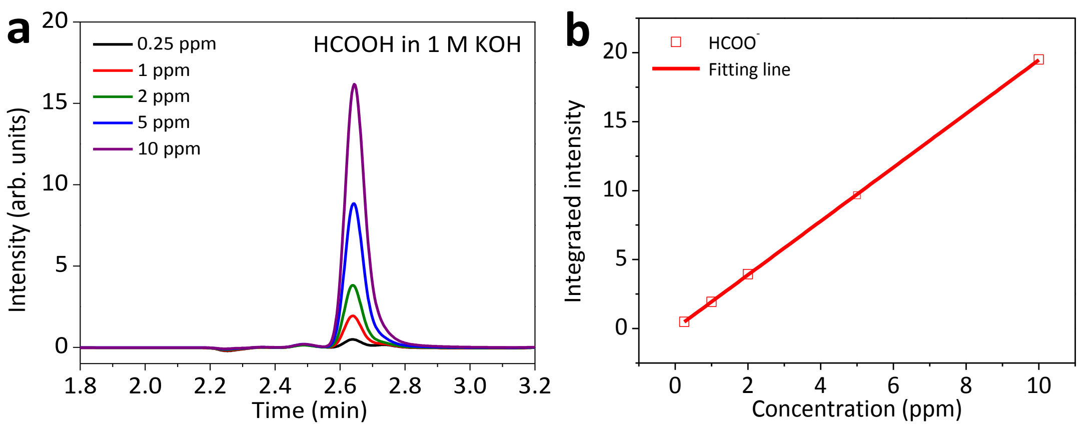

Supplementary Fig. 9. (a) Ion chromatographic (IC) profiles of a series of calibration standard solutions of formate with 1 M KOH. (b) Plot of formate concentration versus integrated density of chromatographic peak.

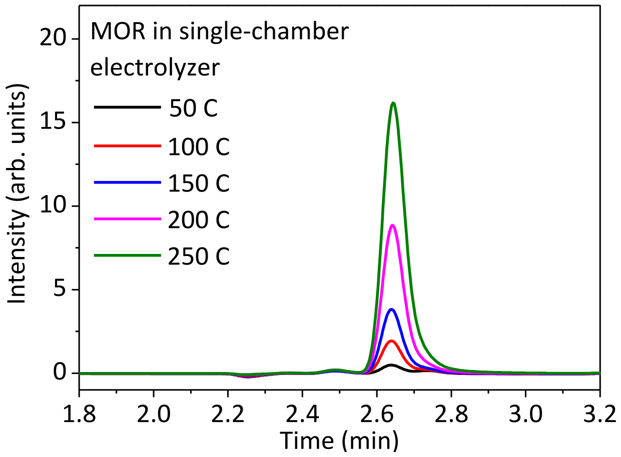

Supplementary Fig. 10. IC profiles of the electrolyte in a single-chamber electrolyzer with 1 M KOH and 3 M CH_3OH at 10 mA cm^{-2} using Pt/C//NiMn-LDH as electrode pair.

Supplementary Table 2. Calculations of formate Faradaic efficiency (%) for the MOR (using single-chamber electrolyzer) on NiMn-LDH based on the IC analysis.

Time (s)	Charge (C)	Produced formate (mmol)	Faradaic efficiency (%)
1000	50	0.132	101.7
2000	100	0.274	105.6
3000	150	0.377	96.8
4000	200	0.497	95.7
5000	250	0.665	102.5

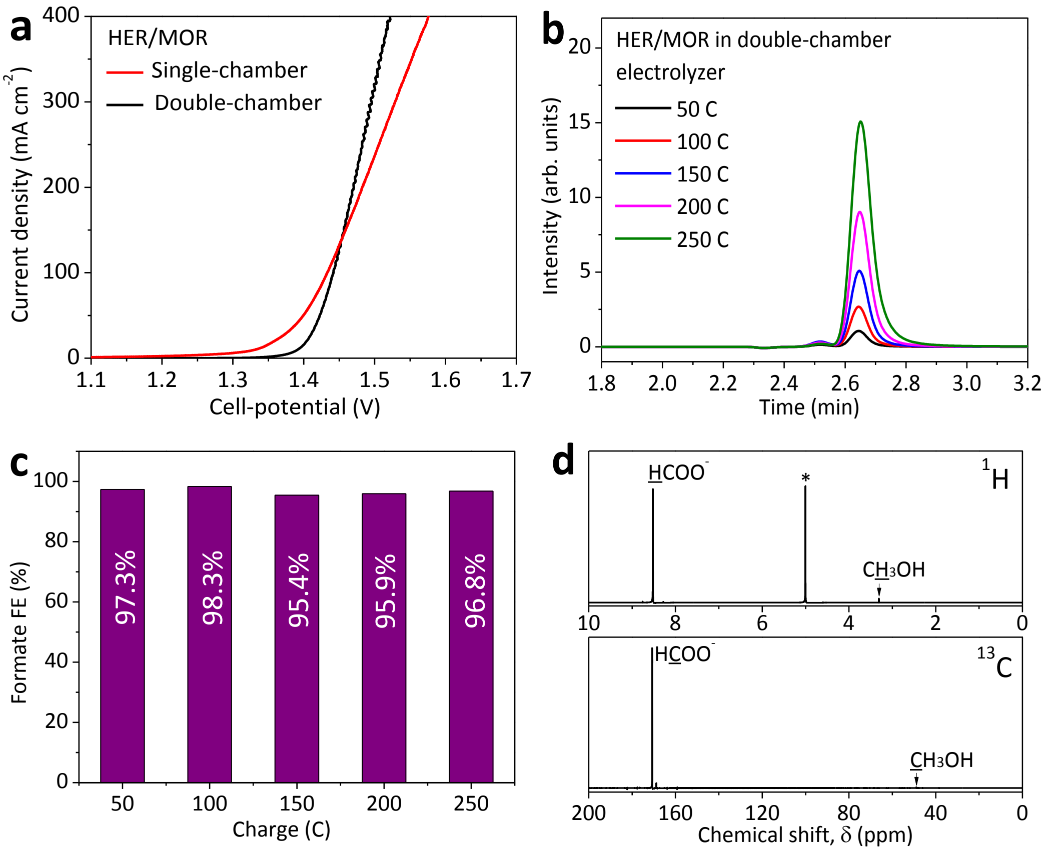

Supplementary Fig. 11. (a) Cell LSV curves of HER/MOR electrolysis using an electrode pair of Pt/C//NiMn-LDH in a single or double-chamber electrolyzer with 1 M KOH and 3 M CH_3OH. (b) IC profiles of the electrolyte in the double-chamber electrolyzer at 10 mA cm^{-2}. (c) Calculated formate Faradaic efficiencies. (d) $^1H/^{13}C$ NMR spectra (400/100 MHz, D_2O) of the electrolyte after a 40-h CP test at 100 mA cm^{-2} using methanol as internal standard. The * marker in ^1H spectrum indicates the signal of H_2O.

Supplementary Table 3. Calculations of formate Faradaic efficiency (%) for the MOR (using double-chamber electrolyzer) on NiMn-LDH based on the IC analysis.

Time (s)	Charge (C)	Produced formate (mmol)	Faradaic efficiency (%)
1000	50	0.126	97.3
2000	100	0.255	98.3
3000	150	0.371	95.4
4000	200	0.501	95.9
5000	250	0.628	96.8

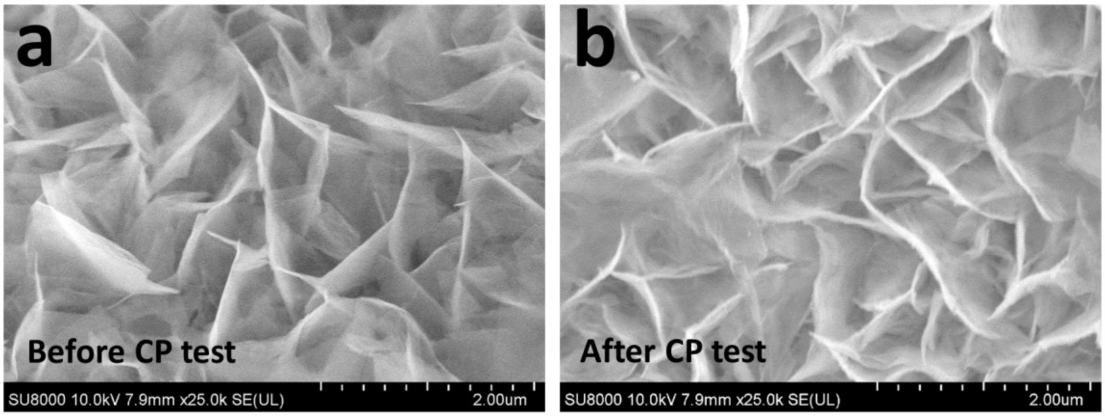

Supplementary Fig. 12. SEM images of NiMn-LDH (a) before and (b) after 20-h CP test at 10 mA cm^{-2} in 1 M KOH with 3 M CH_3OH.

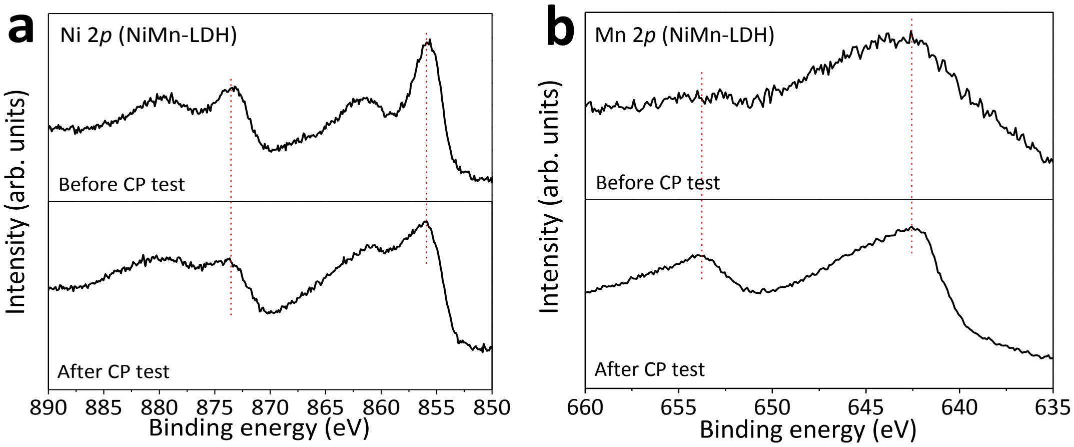

Supplementary Fig. 13. XPS core-level spectra of NiMn-LDH before and after 20-h CP test at 10 mA cm^{-2} in 1 M KOH with 3 M CH_3OH. (a) Ni 2*p* and (b) Mn 2*p*.

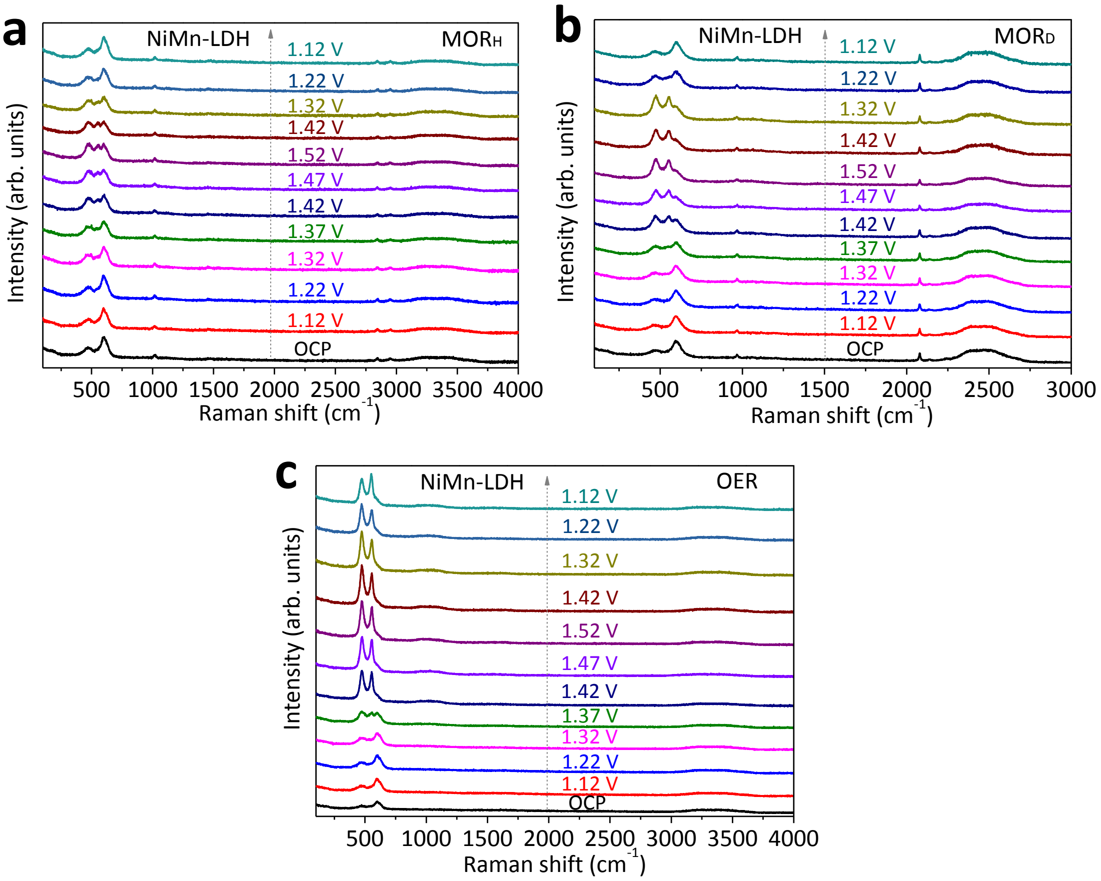

Supplementary Fig. 14. Operando Raman spectra of NiMn-LDH obtained at various potentials under MOR (the subscript H or D denotes CH_3OH/H_2O or CD_3OD/D_2O solution) and OER conditions.

Supplementary note 5

In **Supplementary Fig. 14b, c**, we can observe the broad bands with less or higher intensity in the region of 700-1200 cm^{-1} as the voltage increased to 1.42 V_{RHE}, which is attributed to O-O bond stretching vibration in Ni^{III}-OOH[3,4]. Nevertheless, such broad peaks are very weak in **Supplementary Fig. 14a**, indicating again very limited formation of Ni^{III}-OOH in NiMn-LDH under the normal MOR conditions. Meanwhile, the broad bands in the region of 3100-3600 cm^{-1} in **Supplementary Fig. 14a, c** and those in the region of 2250-2750 cm^{-1} in **Supplementary Fig. 14b** can be assigned to O-H or O-D vibrations of disordered $Ni(OH)_2$ or $Ni(OD)_2$ in NiMn-LDH[3]. In addition, the sharp bands at 1020, 2844 and 2954 cm^{-1} correspond to the methanol, which are all blue-shifted in the deuterated solution.

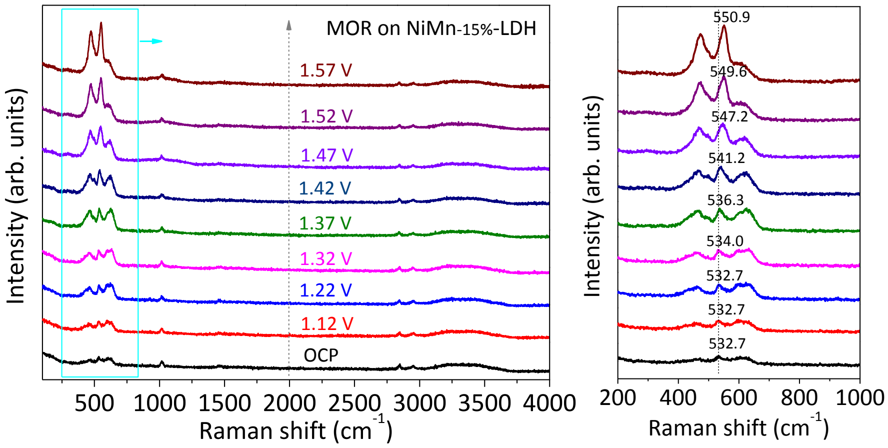

Supplementary Fig. 15. Operando Raman spectra of $Ni_{0.85}Mn_{0.15}$-LDH with more Ni but fewer Mn content obtained at various potentials under MOR conditions.

Supplementary note 6

To prepare $Ni_{0.85}Mn_{0.15}$-LDH, $Ni(NO_3)_2 \cdot 6H_2O$ (0.255 mmol) and $KMnO_4$ (0.045 mmol) were used as precursors. So, as compared to the optimal NiMn-LDH, there is fewer Mn content in $Ni_{0.85}Mn_{0.15}$-LDH. As a result, the operando Raman spectra of $Ni_{0.85}Mn_{0.15}$-LDH in **Supplementary Fig. 15** show more evident bands of Ni^{III}-OOH under the MOR conditions, as compared to **Supplementary Fig. 14**, when the applied potential is increased to 1.37 V_{RHE}. This result further confirms the transient and limited formation of Ni^{III}-OOH in NiMn-LDH under the MOR conditions.

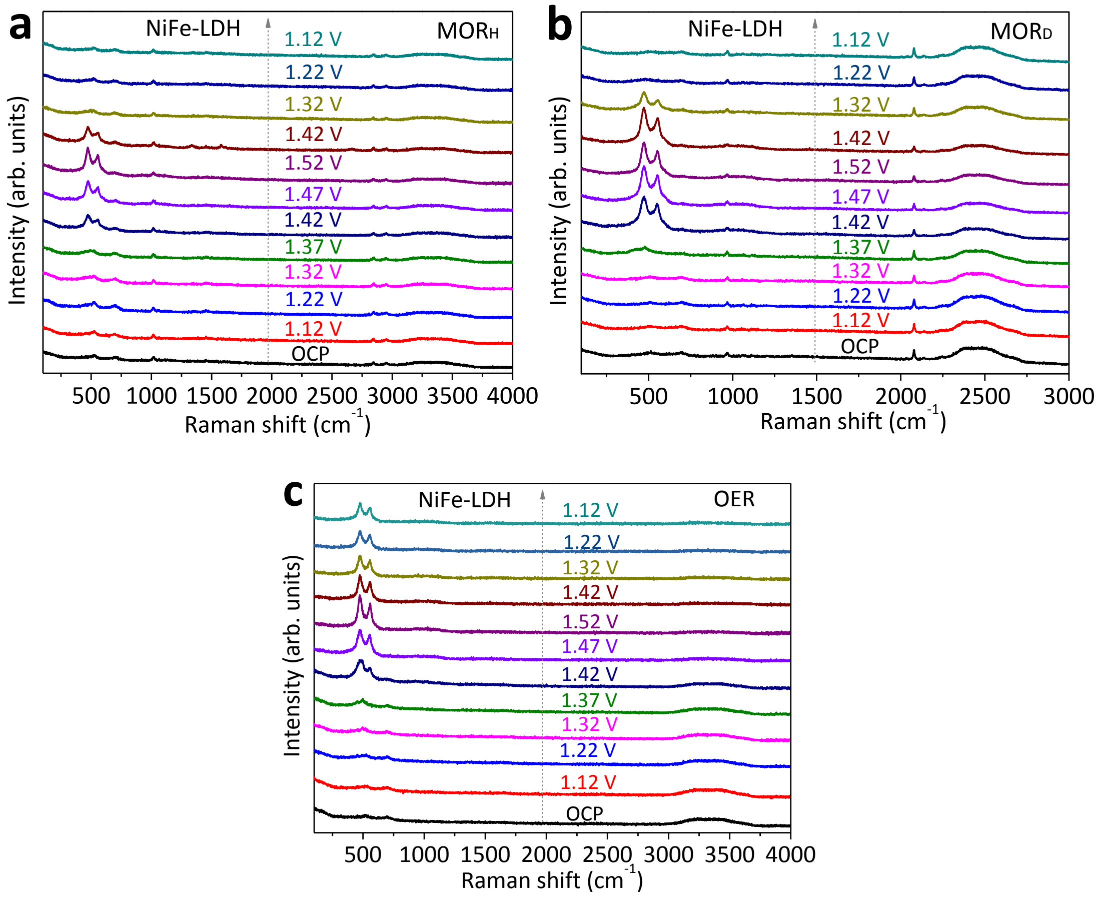

Supplementary Fig. 16. Operando Raman spectra of NiFe-LDH obtained at various potentials under MOR (the subscript H or D denotes CH_3OH (3 M)/H_2O or CD_3OD (3 M)/D_2O solution) and OER conditions.

Supplementary note 7

In **Supplementary Fig. 16**, the bands corresponding to Fe-O stretching vibrations are almost invisible, in line with literature reports[5-7]. Nevertheless, we can observe Raman bands of Ni^{III}-OOH with similar evolution along with the increasing and decreasing of applied potentials under MOR and OER conditions, respectively. This result indicates the transient and limited formation of Ni^{III}-OOH in NiFe-LDH under the normal MOR conditions.

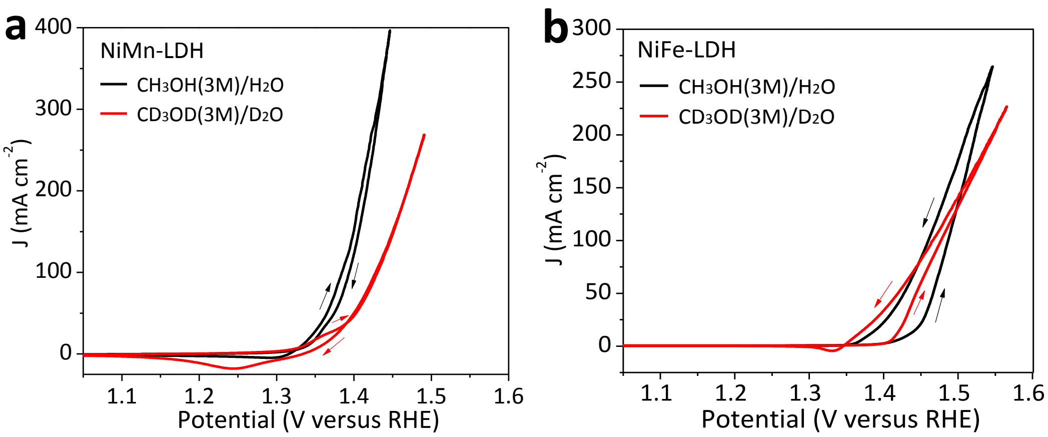

Supplementary Fig. 17. CV curves (with iR compensation) of (a) NiMn and (b) NiFe-LDH in CH_3OH (3 M)/H_2O and CD_3OD (3 M)/D_2O solutions both with 1 M KOH with a scan rate of 5 mV s^{-1}. The arrows indicate the scan orientations.

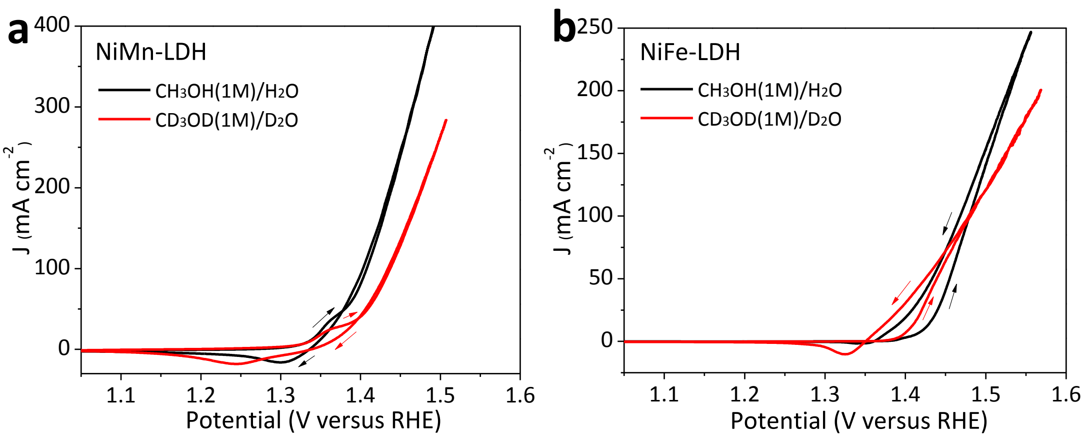

Supplementary Fig. 18. CV curves (with iR compensation) of (a) NiMn and (b) NiFe-LDH in CH_3OH (1 M)/H_2O and CD_3OD (1 M)/D_2O solutions both with 1 M KOH with a scan rate of 10 mV s^{-1}. The arrows indicate the scan orientations.

Supplementary note 8

When methanol concentration is 3.0 M or higher, similar MOR current density could be obtained (see **Supplementary Fig. 34** *vide infra*), indicating that furthering increasing the methanol concentration would not result in enhanced diffusing rate of methanol. When the methanol concentration is decreased from 3.0 to 0.1 M, the MOR current density is gradually decreased, indicating the concentration-dependent diffusing rate of methanol. So, we additionally checked the KIE with 1 M CH_3OH or CD_3OD, where the MOR current density is reduced due to the slower diffusing rate of methanol.

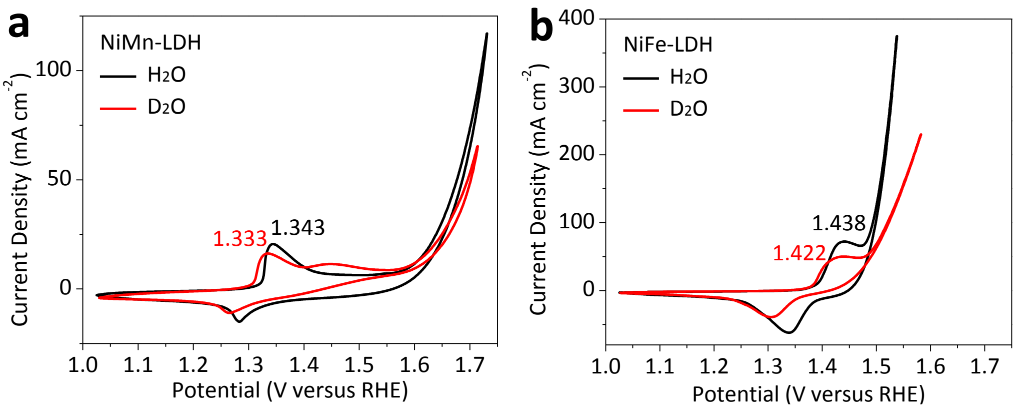

Supplementary Fig. 19. CV curves of (a) NiMn and (b) NiFe-LDH in H_2O and D_2O solutions both with 1 M KOH with a scan rate of 10 mV s^{-1}.

Supplementary note 9

Due to the pseudocapacitive property, NiMn-LDH usually shows broad CV response or that with a small peak-to-peak voltage separation.[8]

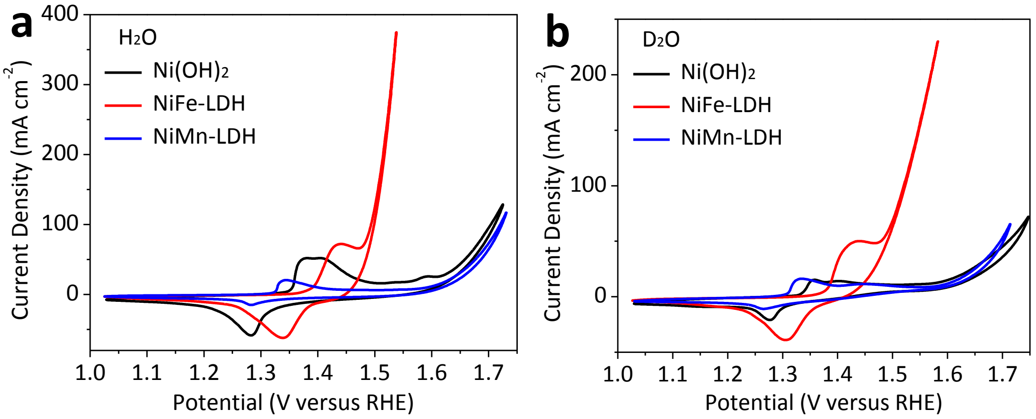

Supplementary Fig. 20. CV curves of as-synthesized NiMn and NiFe-LDH in (a) H_2O and (b) D_2O with 1 M KOH with a scan rate of 10 mV s^{-1}.

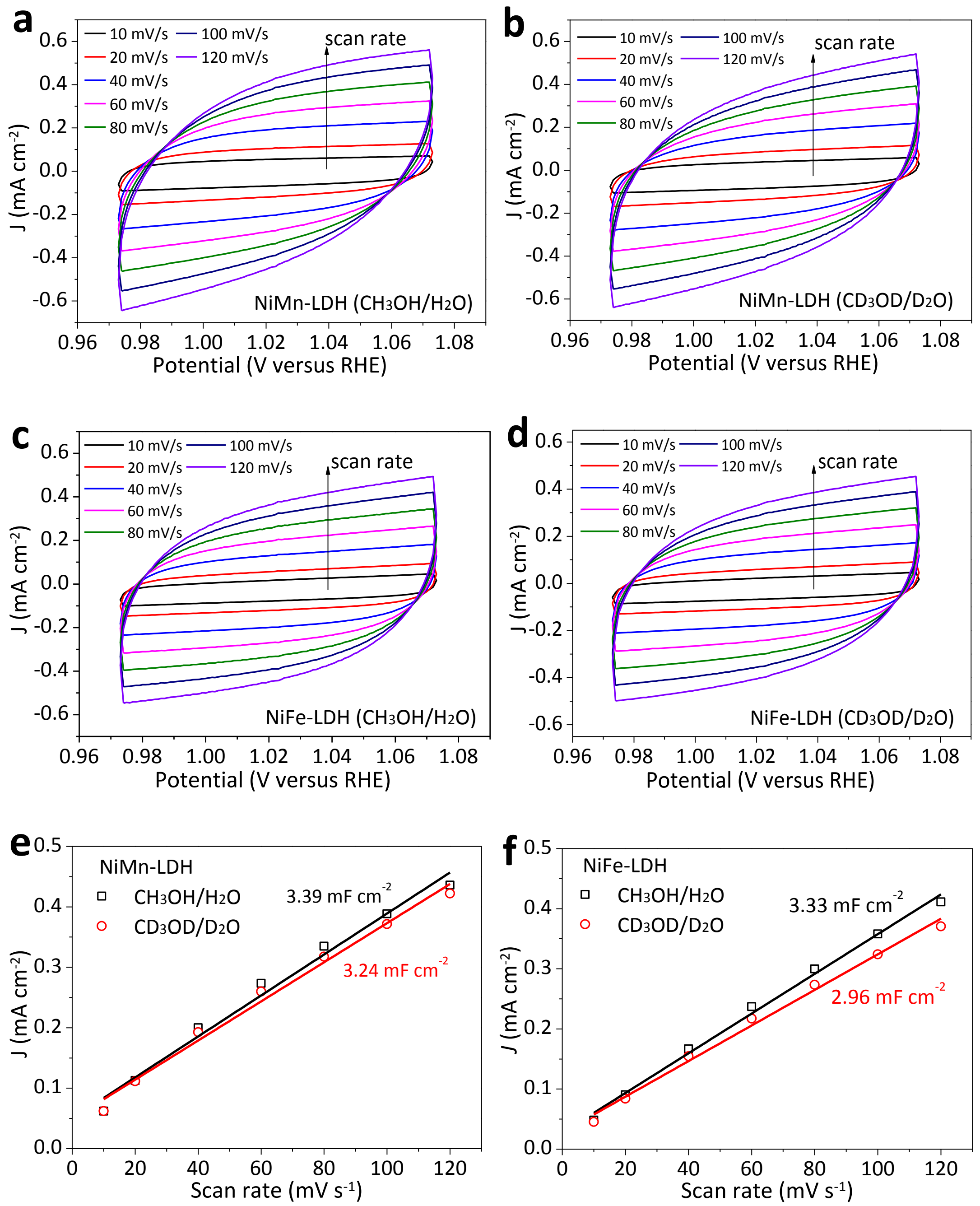

Supplementary Fig. 21. CV curves of (a,b) NiMn-LDH and (c,d) NiFe-LDH measured in the non-Faradaic range in aqueous and deuterated media, respectively. Plots of current density (J) and scan rate (v) for (e) NiMn-LDH and (f) NiFe-LDH.

Supplementary note 10

In the D_2O solution containing 1 M KOH, OD^- is much more dominant than OH^- due to the fast H/D exchange. It has been generally believed that the oxidation of nickel hydroxide in alkaline media is limited by the OH^- adsorption on the catalyst surface. Therefore, the easier Ni^{II}/Ni^{III} oxidation in the deuterium media might be

attributed to the more facile adsorption of OD^- than OH^- on the catalyst surface due to the more polar nature of OD^- than OH^-, which then leaves as water and yields the oxidation of Ni^{II}-$(OH)_2$ to Ni^{III}-OOH.

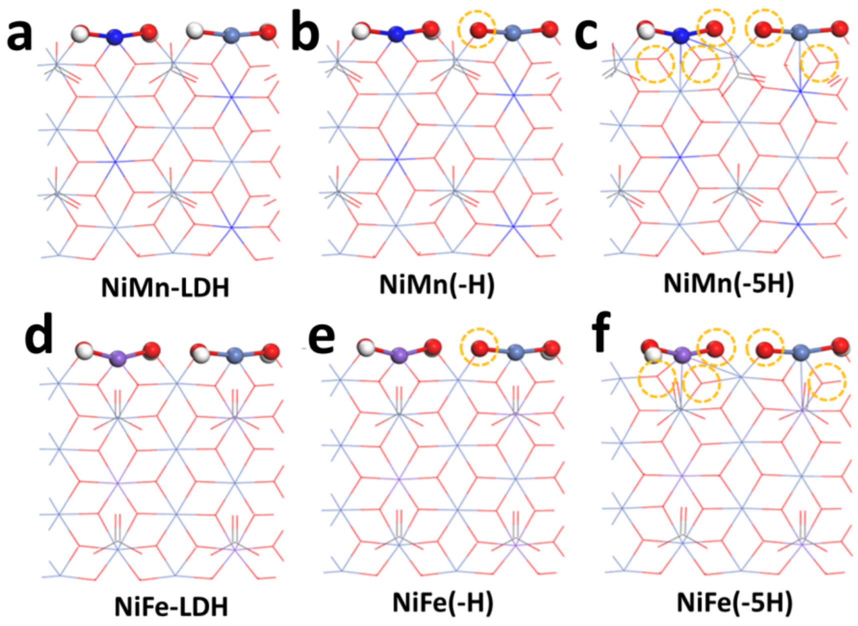

Supplementary Fig. 22. Computation models of NiMn, NiFe-LDH (a,d), NiMn(-H), NiFe(-H) oxyhydroxides for MOR (b,e) and NiMn(-5H), NiFe(-5H) oxyhydroxides for OER (c,f), respectively. Where, the yellow circle indicates the hydrogen-deficient oxygen. Ni: wathet, Fe: purple, Mn: blue, N: gray, O: red, H: white.

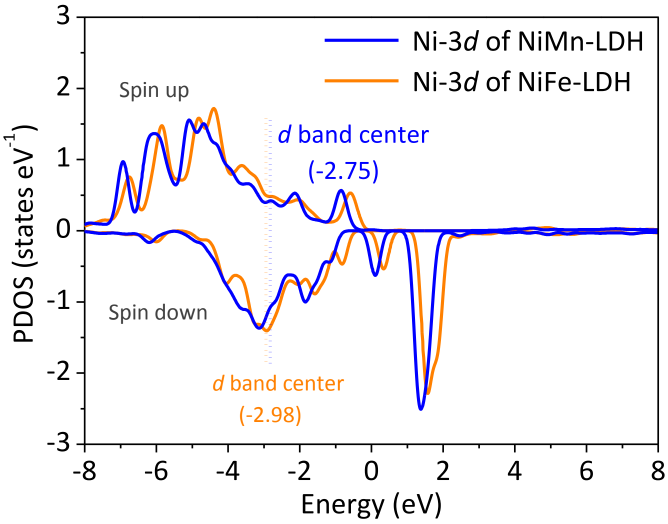

Supplementary Fig. 23 Projected density of states (PDOS) of Ni-3*d* electrons calculated for NiMn and NiFe-LDH.

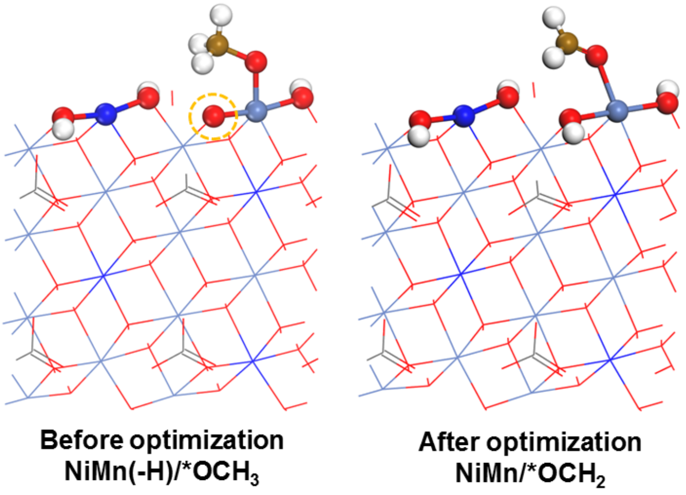

Supplementary Fig. 24. Adsorption structures of NiMn(-H)/*OCH_3 before and after optimization, indicating that NiMn/*OCH_2 rather than NiMn(-H)/*OCH_3 is a stable intermediate.

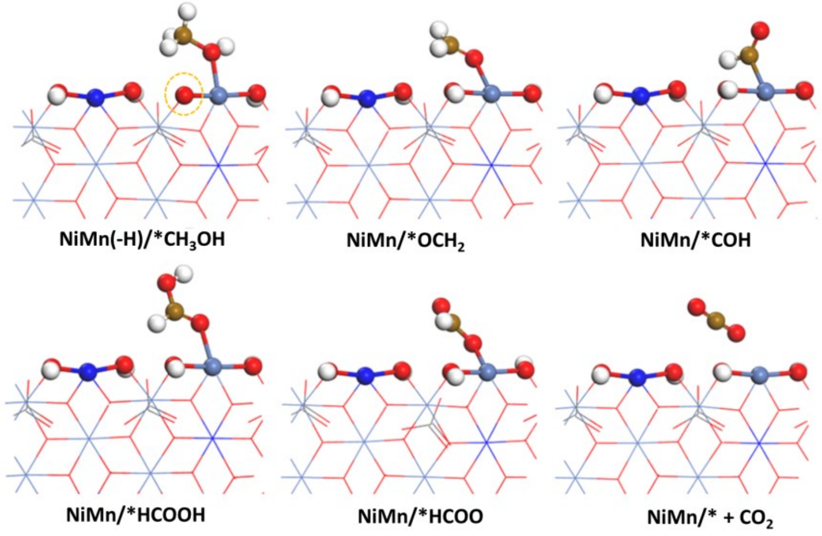

Supplementary Fig. 25. The optimized geometries of adsorbed intermediates for MOR on NiMn(-H). Ni: wathet, Mn: blue, N: gray, O: red, H: white, C: brown. The yellow circle indicates the hydrogen-deficient oxygen in NiMn(-H).

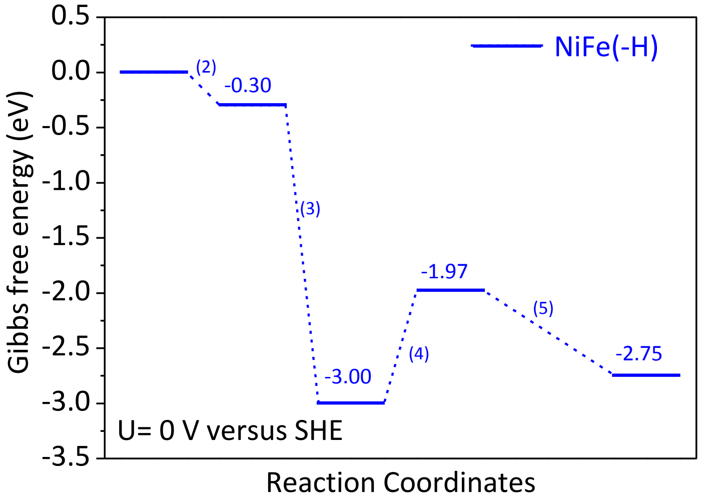

Supplementary Fig. 26. Gibbs free energy diagrams of the MOR on the surface of NiFe(-H) using a same pathway as NiMn(-H).

Supplementary note 11

For the MOR process of NiFe(-H), the same bifunctional mechanism as NiMn(-H) was first considered, and the calculation results are shown in **Supplementary Fig. 26**. Unfortunately, reactions 2, 3 and 5 are exothermic, but reaction 4 is extremely endothermic with $\Delta G = 1.03$ eV, which is not consistent with the experimental observation (KIE results). Consequently, we propose that, following the O-H dehydrogenation, the reduction of NiFe(-H) to NiFe occurs along with the double C-H dehydrogenation of methanol.

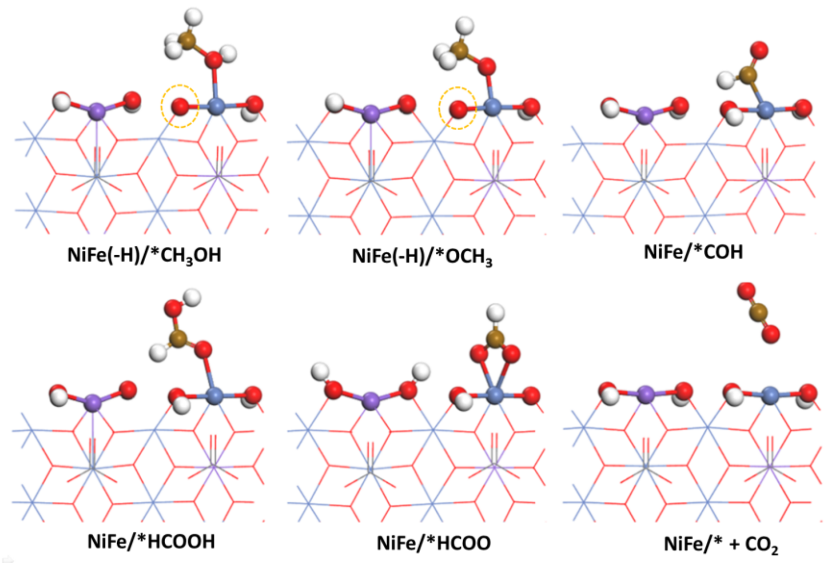

Supplementary Fig. 27. The optimized geometries of adsorbed intermediates for MOR on NiFe(-H). Ni: wathet, Fe: purple, N: gray, O: red, H: white, C: brown. The yellow circle indicates the hydrogen-deficient oxygen in NiFe(-H).

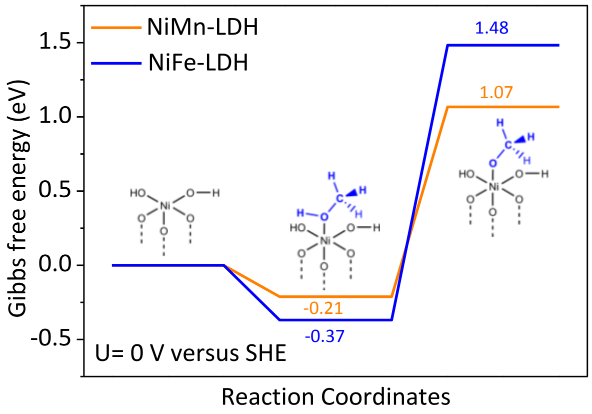

Supplementary Fig. 28. Gibbs free energy diagrams of the MOR on the surface of NiMn and NiFe-LDH.

Supplementary note 12

It can be seen that the dehydrogenation step from NiM/*CH_3OH to NiM/*OCH_3 are both thermodynamically unfavorable on the surface of NiMn and NiFe-LDH due to the high uphill energy barrier, which contradicts with KIE observations that the catalytic MOR on NiFe-LDH is spontaneous. So, NiMn and NiFe-LDH are not likely to be the real catalyst for the catalytic MOR process.

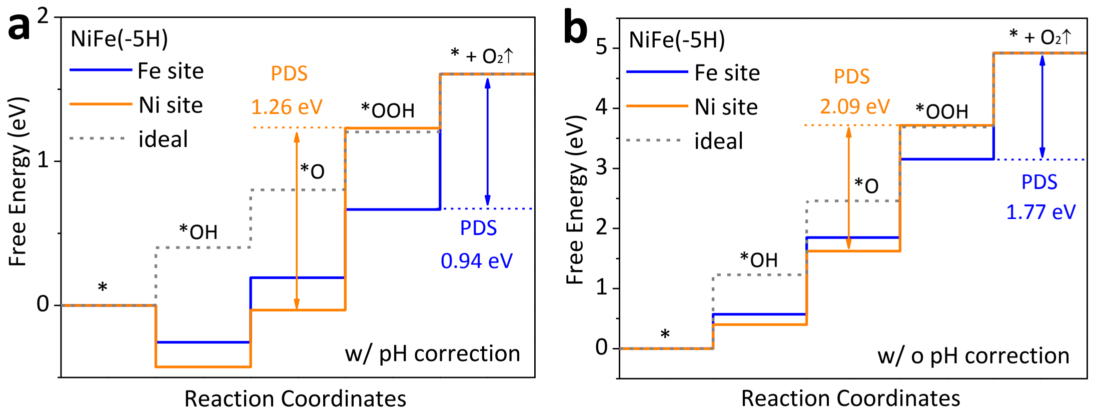

Supplementary Fig. 29. Gibbs free energy diagram for OER on the Ni and Fe sites of NiFe(-5H) (a) with and (b) without pH correction, respectively.

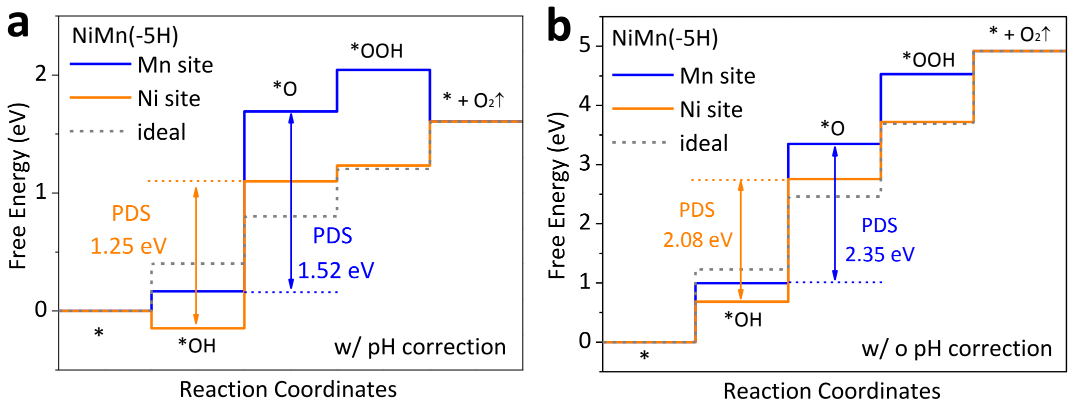

Supplementary Fig. 30. Gibbs free energy diagram for OER on Ni and Mn sites of NiMn(-5H) (a) with and (b) without pH correction, respectively.

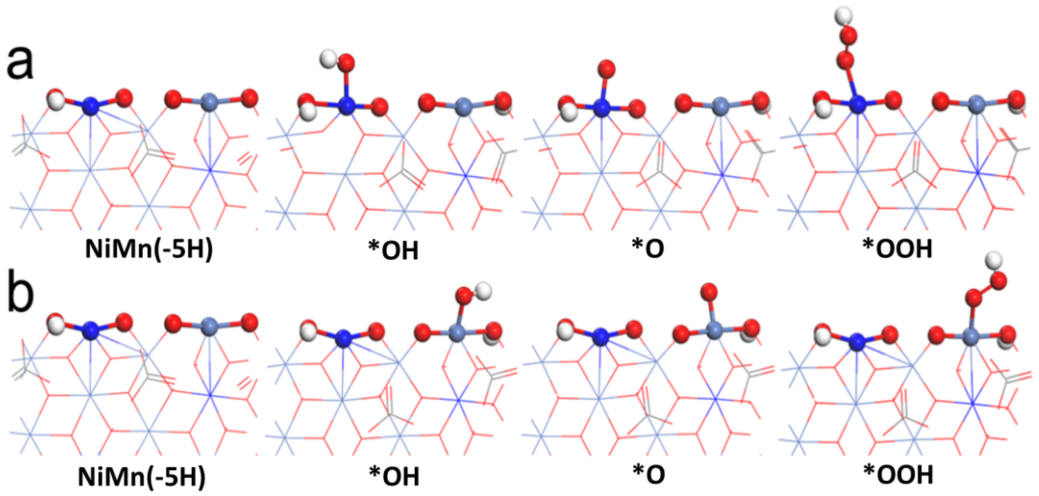

Supplementary Fig. 31. The optimized geometries of adsorbed intermediate of OER for (a) Mn and (b) Ni sites of NiMn(-5H) oxyhydroxide, respectively.

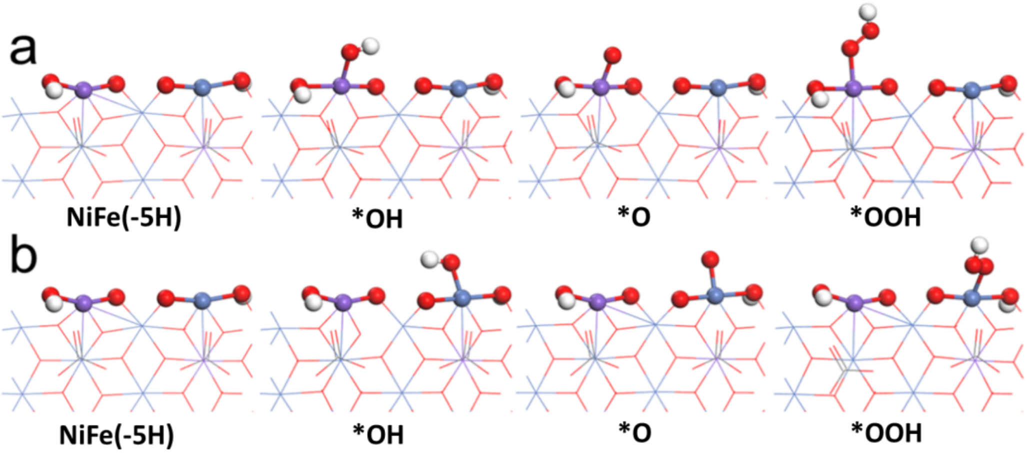

Supplementary Fig. 32. The optimized geometries of adsorbed intermediate of OER for (a) Fe and (b) Ni sites of NiFe(-5H) oxyhydroxide, respectively.

Supplementary note 13

To verify the feasibility of the DFT models and methods applied in this study, we also conducted computations for OER that has been well studied in the literature works.[9] It has been well established that NiM oxyhydroxide rather than NiM-LDH is the origin of OER. In our experiments, we also observed dominant formation of NiM oxyhydroxide before and during the OER, in line with the literature report.[10] Therefore, a four-layered NiM-LDH with partially removed hydrogen atoms (the model is denoted as NiM(-5H), as shown in **Supplementary Fig. 22c, f**) was adopted as the computational model for the OER, in which all exposed metal sites were explored to determine active site. The computation results are summarized in **Supplementary Fig. 29-32**. For the NiMn(-5H) model, the dehydrogenation of *OH to form *O (* denotes Mn or Ni-site) is determined as PDS with the largest Gibbs free energy barrier (ΔG) of 1.52/1.25 eV (Mn/Ni-site, with pH correction). Whereas, for the NiFe(-5H) model, the *OOH→O_2 + * and *O→*OOH steps are considered as PDS of Fe and Ni-site due to the largest ΔG of 0.94/1.26 eV (Fe/Ni-site, with pH correction), respectively. Thus, the real active site for the OER on NiMn(-5H) model is Ni-site, while that for NiFe(-5H) model is Fe-site. As the PDS of NiMn(-5H) needs to overcome a larger energy barrier than that of NiFe(-5H), the computation results suggest that the NiMn(-5H) model shall require a higher potential to trigger the OER, in line with the experimental results and literature report.[11]

Supplementary note 14

Materials: Iron(III) nitrate nonahydrate ($Fe(NO_3)_3 \cdot 9H_2O$, 99.99%), deuterium oxide (99.9% atom% D), Methanol-D4 (99.8% atom% D) and potassium hydroxide (KOH, 99.99%) were purchased from Innochem Co. Ltd. Nickel(II) nitrate hexahydrate (($Ni(NO_3)_2 \cdot 6H_2O$, ≥98.0%), potassium permanganate ($KMnO_4$, 99.5%), urea (≥99.0%), methanol (99%) and formate acid (99%) were from Sinopharm Chemical Reagent Co. Ltd (Shanghai, China). Nafion solution (5 wt%) was from Alfa Aesar, while Pt/C (20 wt%) and RuO_2 were from J&K Scientific Co. Deionized water with a resistivity ≥18 MΩ was supplied from a Millipore-Q system.

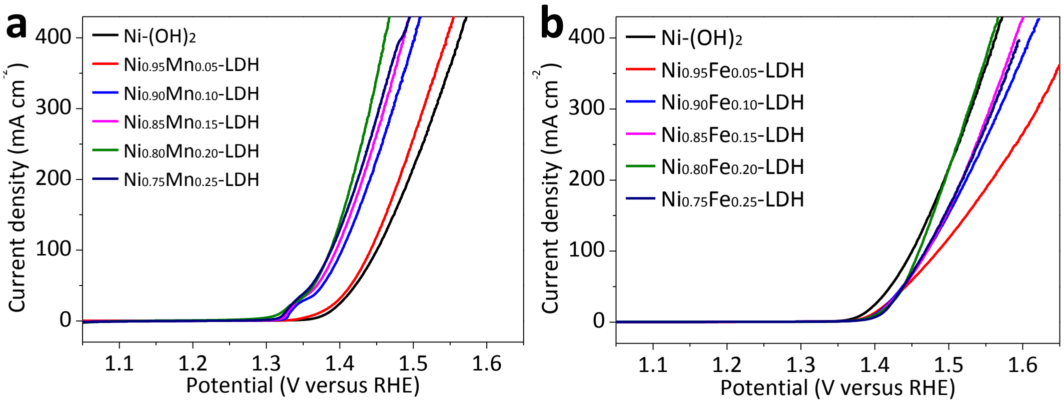

Supplementary Fig. 33. LSV curves (with iR compensation) of a series of (a,b) $Ni_{1-y}Mn_y$-LDH and (c,d) $Ni_{1-y}Fe_y$-LDH under the MOR conditions (3 M CH_3OH and 1 M KOH). $Ni(OH)_2$ nano-catalyst synthesized using the same method is used as reference.

Supplementary note 15

To optimize the M content in NiM-LDH (M = Mn, Fe), we synthesized a series of $Ni_{1-y}M_y$-LDH, where y represents the relative molar content of M^{x+} precursor. Their LSV curves were recorded under the MOR conditions (3 M CH_3OH and 1 M KOH). As shown in the **Supplementary Fig. 33a**, the $Ni_{0.80}Mn_{0.20}$-LDH performs best, followed by $Ni_{0.75}Mn_{0.25}$, $Ni_{0.85}Mn_{0.15}$, $Ni_{0.90}Mn_{0.10}$ and $Ni_{0.95}Mn_{0.05}$-LDH. As for NiFe-LDH (**Supplementary Fig. 33b)**, $Ni_{0.75}Fe_{0.25}$ and $Ni_{0.80}Fe_{0.20}$-LDH show very comparable performance, followed by $Ni_{0.85}Fe_{0.15}$, $Ni_{0.90}Fe_{0.10}$ and $Ni_{0.95}Fe_{0.05}$-LDH. So, in this work, we focused on the best performed $Ni_{0.80}Mn_{0.20}$ and $Ni_{0.80}Fe_{0.20}$-LDH,

both of which were synthesized with the same precursors ratio. For simplicity, $Ni_{0.80}Mn_{0.20}$ and $Ni_{0.80}Fe_{0.20}$-LDH are denoted as NiMn and NiFe-LDH in the main text.

Supplementary note 16

Electrochemical measurements: The electrochemical measurements were performed using a standard three-electrode system connected to a CHI 760E electrochemical workstation (Chen Hua, China) at room temperature. The as-prepared samples (0.5×1 cm^2), graphite rod and Ag/AgCl (with salt bridge) were used as the working, counter and reference electrodes, respectively. A PTFE-made cell was used for holding the alkaline electrolyte and three electrodes. Before the electrochemical tests, the cyclic voltammetry (CV) was tested until a constant curve was achieved. The CV activations for all catalysts were conducted at a scan rate of 50 mV s^{-1} in the potential range of 1.02-1.62 V_{RHE} in corresponding electrolyte.

Linear sweep voltammetry (LSV) curves of OER and MOR were measured in 1 M KOH solution without and with and 3 M methanol with a scan rate of 5 mV s^{-1}. As shown in **Supplementary Fig. 34**, the catalyst NiMn-LDH shows the best MOR activity with 3.0, 3.5 and 4 M methanol. So, all MOR tests in this work were performed with 3 M methanol to achieve the best cost performance.

To perform HER/MOR electrolysis, a two-electrode system was set up by using a single-chamber electrolyzer or a double-chamber electrolyzer (H-cell) with a Nafion 117 membrane as separator. Before setting up, the Nafion membrane was first treated in 5 wt% hydrogen peroxide at 80 °C for 1 h, then soaked in deionized water for 30 min; and then boiled in 5 wt% dilute sulfuric acid at 80 °C for 1 h; and finally soaked in deionized water for 30 min.

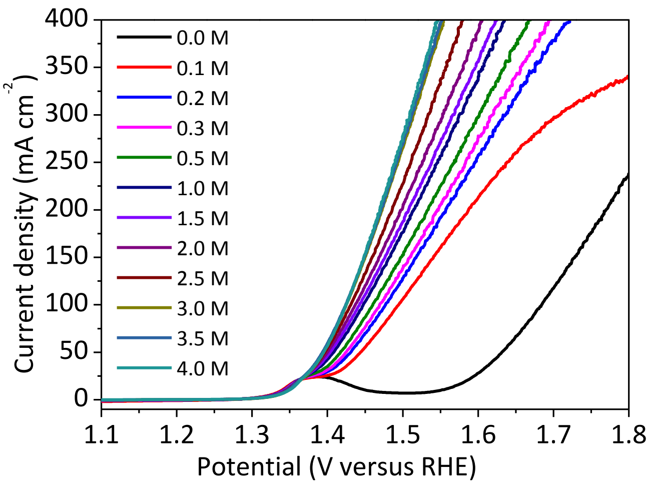

Supplementary Fig. 34. LSV curves (with iR compensation) of as-synthesized NiMn-LDH in 1 M KOH with different methanol concentrations with a scan rate of 5 mV s^{-1}.

All potentials data standardized versus the RHE convert from the following equation: $E_{(RHE)} = E_{(Ag/AgCl)} + 0.197 + 0.0591 \times pH$. In addition, for all LSV curves, 90% iR drop compensation was applied manually after the experiment, where R was notarized by fitting electrochemical impedance spectroscopy (EIS) data. For instance, the Nyquist plots derived from the EIS measurements in CH_3OH(3M)/H_2O and CD_3OD(3M)/D_2O with 1 M KOH are provided as follows.

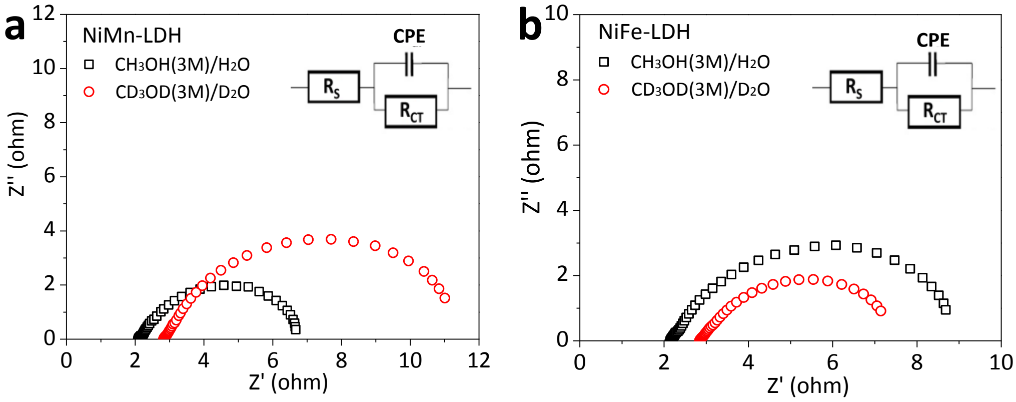

Supplementary Fig. 35. Nyquist plots were obtained from EIS measurements for (a) NiMn and (b) NiFe-LDH (both grown on NF) under the MOR conditions (3 M CH_3OH or CD_3OD and 1 M KOH). Inset shows the equivalent circuit, in which R_S corresponds to the internal resistance of electrolyte solution. The data show that the internal resistance is increased by 0.7 ohm by using deuterium media to replace aqueous media.

The electrochemical active surface area (ECSA) was evaluated by the equation of ECSA = C_{dl}/C_s, where C_{dl} and C_s refer to the double-layer capacitance and specific capacitance, respectively.[12] The C_{dl} of the catalyst was measured by means of scanning rate-dependent CV in a potential range of non-faradic district (about 0.97-1.07 V_{RHE}. As the C_s may change depending on the composition of the electrocatalyst, it is determined from independent experiment utilizing known-area electrode according to the following equation[13]:

$$C_s = \frac{\int_{V_1}^{V_2} I\, dV}{2v\, \Delta V} \tag{1}$$

Where $\int_{V_1}^{V_2} I\, dV$ is the area of CVs, v is scan rate (V s^{-1}), and ΔV is potential window. Thus, C_s is the slope of scan rate and $\frac{\int_{V_1}^{V_2} I\, dV}{2\Delta V}$ plot.

To perform electrochemical measurements with deuterated media, we prepared D_2O solution containing 3 M CD_3OD and 1 M KOH. To reach equilibrium between D_2O and KOH, the anode was immersed in the deuterated media for 10 min before electrochemical measurement.

Supplementary note 17

Calculation method for FE: The generated formate at the anode was detected by ion chromatography (IC). The Faraday efficiency (FE) of formate can be calculated using the following equation:

$$\mathrm{FE(formate)} = \frac{\mathrm{n} \times 4 \times \mathrm{N_A} \times \mathrm{e}}{\mathrm{Q}} \times 100\% \tag{2}$$

where n is the mol of generated formate; 4 is the number of transferred electrons; N_A is Avogadro constant (6.02×10^{23} mol^{-1}); e is elementary charge (1.60×10^{-19} C); Q is the passed charge (C).

Supplementary note 18

Computational Methods: Spin-polarized density functional theory (DFT) + U calculations were performed via the Vienna ab-initio Simulation Package (VASP)[14] with the projector augmented wave (PAW) method[15]. A U-J value of 3.8 for Ni, 3.5 for Mn and 4.3 for Fe were used to describe electron correlation for 3d metals (Ni, Fe

and Mn in this work according to the literatures[16-18]. Generalized gradient approximation (GGA) parameterized by Perdew-Burke-Ernzerhof (PBE) for exchange-correlation functional was chosen for all the calculations[19]. Grimme's correction method (DFT-D3) was used to describe the van der Waals (vdW) interactions between catalyst and adsorbate[20]. The periodic slab model was optimized using the Monkhorst-Pack method with a k-point of 3 × 2 × 1 for electronic structure calculation with a cutoff energy of 450 eV. The ionic and electronic degrees of freedom were converged within 10^{-5} eV for the energy and 0.03 eV $Å^{-1}$ for the forces on per atom. A vacuum space of 16 Å was added to the vertical direction to minimize the interactions between periodic images.

To better match the experimental results, the Ni/M atomic ratio of NiM-LDH model (M = Mn, Fe) is set as 3:1. In addition, a four-layered NiM-LDH model with (110) surface and with the termination of unsaturated metal sites were constructed[21].

(1). MOR computational methods:

To assess the one-electron oxidation of NiM to NiM(-H), the reaction energy (ΔE) of this process was calculated similar to HER:

$$\Delta E_{(1)} = [E(\mathrm{NiM(-H)})] - [E(\mathrm{NiM}) - E(\mathrm{H_2})/2] \quad (3)$$

Where the subscript (1) corresponds to the reaction (1) in the manuscript. The computational hydrogen electrode (CHE) model was applied to calculate the reaction energy involving the proton-electron transfer.

In addition, to probe the reaction intermediates, the reaction energies were defined as listed below:

$$\Delta E_{(2)} = [E(\mathrm{NiM(-H)/{*}CH_3OH})] - [E(\mathrm{NiM(-H)}) + E(\mathrm{CH_3OH})] \quad (4)$$

$$\Delta E_{(3)} = [E(\mathrm{NiM/{*}OCH_2}) + E(\mathrm{H_2})/2] - [E(\mathrm{NiM(-H)/{*}CH_3OH})] \quad (5)$$

$$\Delta E_{(4)} = [E(\mathrm{NiM/{*}COH}) + E(\mathrm{H_2})/2] - [E(\mathrm{NiM/{*}OCH_2})] \quad (6)$$

$$\Delta E_{(5)} = [E(\mathrm{NiM/{*}HCOOH}) + E(\mathrm{H_2})/2] - [E(\mathrm{NiM/{*}COH}) + E(\mathrm{H_2O})] \quad (7)$$

$$\Delta E_{(6)} = [E(\mathrm{NiM/{*}HCOO}) + E(\mathrm{H_2})/2] - [E(\mathrm{NiM/{*}HCOOH})] \quad (8)$$

$$\Delta E_{(7)} = [E(\mathrm{NiM/{*}COO}) + E(\mathrm{H_2})/2] - [E(\mathrm{NiM/{*}HCOO})] \quad (9)$$

Where E(NiM(-H)), E(CH_3OH), E(H_2O) and E(H_2) denote the total energies of bare surface of catalyst, CH_3OH, H_2O and H_2, respectively. The energy of OH^- is converted by the equation as follow: $H_2O = H^+ + OH^-$. It is worth noting that the ΔE of step 3′ and 4′ of NiFe-LDH are calculated using the following equation:

$$\Delta E_{(3')} = [E(\text{NiM(-H)/*OCH}_3) + E(\text{H}_2)/2] - [E(\text{NiM(-H)/*CH}_3\text{OH})] \quad (10)$$

$$\Delta E_{(4')} = [E(\text{NiM/*COH}) + E(\text{H}_2)/2] - [E(\text{NiM(-H)/*OCH}_3)] \quad (11)$$

The Gibbs free energy for the adsorption of intermediates can be calculated by:

$$\Delta G = \Delta E + \Delta ZPE - T\Delta S - neU \quad (12)$$

Where ΔE is the energy change of the reaction step based on DFT calculations, T is temperature (298.15 K), ΔZPE and ΔS are the contributions to variation in zero-point energy and entropy, respectively, and n is the electron number of such state, U is an applied electrode potential.

(2). OER computation methods:

To perform DFT computations for the OER on NiM-LDH, the same NiM-LDH models as those for MOR are adopted. Nevertheless, unlike the very limited and reversible formation of oxyhydroxide species (Ni(M)OOH) under the MOR conditions, the Ni(M)OOH formation is dominant under the OER conditions. In other words, once the Ni(M)OOH is formed, it remains stable under the OER conditions, as suggested by literatures and our operando Raman studies. So, to construct an oxidized model, five hydrogen atoms are removed from the top surface of pristine NiM-LDH to represent the dominant formation of Ni(M)OOH under the OER conditions, which is denoted as NiM(-5H) (M = Mn, Fe).

It has been well established that the alkaline OER generally proceeds through the four-step associative mechanism. All the elementary steps are listed below as reactions ($1^\dagger$)-($4^\dagger$).

$$OH^- + * \rightarrow *OH + e^- \quad (1^\dagger)$$

$$*OH + OH^- \rightarrow *O + H_2O(l) + e^- \quad (2^\dagger)$$

$$*O + OH^- \rightarrow *OOH + e^- \quad (3^\dagger)$$

$$*OOH + OH^- \rightarrow O_2(g) + H_2O(l) + e^- + * \quad (4^\dagger)$$

Total reaction is listed below as reaction (5†):

$$4OH^{-} \rightarrow O_2(g) + 2H_2O(l) + 4e^{-} \quad (5^\dagger)$$

where * represents active site on the surface of catalyst. The *OH, *O, *OOH represented the intermediates during OER.

The Gibbs free energies change could then be obtained similar to MOR:

$$\Delta G_1 = E(*OH) + E(H_2)/2 - E(*) - E(H_2O) + \Delta ZPE - T\Delta S - eU + \mathbf{k_B}T\ln 10\times pH \quad (6^\dagger)$$

$$\Delta G_2 = E(*O) + E(H_2)/2 - E(*OH) + \Delta ZPE - T\Delta S - eU + \mathbf{k_B}T\ln 10\times pH \quad (7^\dagger)$$

$$\Delta G_3 = E(*OOH) + E(H_2)/2 - E(*O) - E(H_2O) + \Delta ZPE - T\Delta S - eU + \mathbf{k_B}T\ln 10\times pH \quad (8^\dagger)$$

$$\Delta G_4 = 1.604 - \Delta G_1 - \Delta G_2 - \Delta G_3 \quad (9^\dagger)$$

Where the $\mathbf{k_B}$ is Boltzman constant and pH is 14.00 in our system. The equilibrium potential for OER was determined to be 0.401 V vs SHE in alkaline media. ΔG_4 was calculated by $1.604 - \Delta G_1 - \Delta G_2 - \Delta G_3$ to avoid calculating the energy of O_2.

Supplementary References

1. Chala SA, et al. Hierarchical 3D architectured Ag nanowires shelled with NiMn-layered double hydroxide as an efficient bifunctional oxygen electrocatalyst. *ACS Nano* **14**, 1770-1782 (2020).
2. Yu L, et al. Cu nanowires shelled with NiFe layered double hydroxide nanosheets as bifunctional electrocatalysts for overall water splitting. *Energy Environ. Sci.* **10**, 1820-1827 (2017).
3. Chala SA, et al. Tuning dynamically formed active phases and catalytic mechanisms of in situ electrochemically activated layered double hydroxide for oxygen evolution reaction. *ACS Nano* **15**, 14996-15006 (2021).
4. Qi Y, et al. Insights into the activity of nickel boride/nickel heterostructures for efficient methanol electrooxidation. *Nat. Commun.* **13**, 4602 (2022).
5. Zhang W-D, et al. In-situ generated Ni-MOF/LDH heterostructures with abundant phase interfaces for enhanced oxygen evolution reaction. *Appl. Catal. B: Environ.* **286**, 119906 (2021).
6. Louie MW, Bell AT. An investigation of thin-film Ni-Fe oxide catalysts for the electrochemical evolution of oxygen. *J. Am. Chem. Soc.* **135**, 12329-12337 (2013).
7. Bai L, Lee S, Hu X. Spectroscopic and electrokinetic evidence for a bifunctional mechanism of the oxygen evolution reaction. *Angew. Chem., Int. Ed.* **60**, 3095-3103 (2021).
8. Tang Y, et al. Fabrication of oxygen-vacancy abundant NiMn-layered double hydroxides for ultrahigh capacity supercapacitors. *Adv. Funct. Mater.* **30**, 1908223 (2020).
9. Wang B, et al. Structure inheritance strategy from MOF to edge-enriched NiFe-LDH array for enhanced oxygen evolution reaction. *Appl. Catal. B: Environ.* **298**, 120580 (2021).
10. Dionigi F, et al. In-situ structure and catalytic mechanism of NiFe and CoFe layered double hydroxides during oxygen evolution. *Nat. Commun.* **11**, 2522 (2020).
11. Dionigi F, et al. Intrinsic electrocatalytic activity for oxygen evolution of crystalline 3d-transition metal layered double hydroxides. *Angew. Chem., Int. Ed.* **60**, 14446-14457 (2021).
12. Liu G, et al. 3D porous network heterostructure NiCe@NiFe electrocatalyst for efficient oxygen evolution reaction at large current densities. *Appl. Catal. B: Environ.* **260**, 118199 (2020).
13. Liu C, Hirohara M, Maekawa T, Chang R, Hayashi T, Chiang C-Y. Selective electro-oxidation of glycerol to dihydroxyacetone by a non-precious electrocatalyst - CuO. *Appl. Catal. B: Environ.* **265**, 118543 (2020).
14. Kresse G, Furthmüller J. Efficient iterative schemes for ab initio total-energy calculations using a plane-wave basis set. *Phys. Rev. B* **54**, 11169-11186 (1996).
15. Blöchl PE. Projector augmented-wave method. *Phys. Rev. B* **50**, 17953-17979 (1994).
16. Dudarev SL, Botton GA, Savrasov SY, Humphreys CJ, Sutton AP. Electron-energy-loss spectra and the structural stability of nickel oxide: An LSDA+U study. *Phys. Rev. B* **57**, 1505-1509 (1998).
17. Kulkarni A, Siahrostami S, Patel A, Nørskov JK. Understanding catalytic activity trends in the oxygen reduction reaction. *Chem. Rev.* **118**, 2302-2312 (2018).
18. Fan K, et al. Nickel-vanadium monolayer double hydroxide for efficient electrochemical water oxidation. *Nat. Commun.* **7**, 11981 (2016).

19. Perdew JP, Burke K, Ernzerhof M. Generalized gradient approximation made simple. *Phys. Rev. Lett.* **77**, 3865-3868 (1996).
20. Grimme S, Antony J, Ehrlich S, Krieg H. A consistent and accurate ab initio parametrization of density functional dispersion correction (DFT-D) for the 94 elements H-Pu. *J. Chem. Phys.* **132**, 154104 (2010).
21. Bi Y, et al. Understanding the incorporating effect of Co^{2+}/Co^{3+} in NiFe-layered double hydroxide for electrocatalytic oxygen evolution reaction. *J. Catal.* **358**, 100-107 (2018).
