# Supplementary material for: Unraveling a bifunctional mechanism for methanol-to-formate electro-oxidation on nickel-based hydroxides
Source: Nat Commun. 2023 Mar 27;14:1686. doi: 10.1038/s41467-023-37441-9 (PMC10042884; doi:10.1038/s41467-023-37441-9)
Supplement: Supplementary file 3 — Description of Additional Supplementary Files [file 41467_2023_37441_MOESM3_ESM.pdf]

Description of Additional Supplementary Files

File Name: Supplementary Movie 1
Description: The video shows the color change of NiFe-LDH-based anode during various LSV tests, which were performed under the OER and MOR conditions, respectively. Up: OER with 1M KOH; Middle: MOR with 1M KOH and 3M CH_3OH; Bottom: MOR with 1M KOH and 3M CD_3OD in D_2O solution.

File Name: Supplementary Movie 2
Description: The video shows the color change of NiMn-LDH-based anode during various LSV tests, which were performed under the OER and MOR conditions, respectively. Up: OER with 1M KOH; Middle: MOR with 1M KOH and 3M CH_3OH; Bottom: MOR with 1M KOH and 3M CD_3OD in D_2O solution.

File Name: Source data
Description: The experimental data generated in this study.
